# A case of superior mesenteric artery syndrome got physicians in trouble

**DOI:** 10.1093/jscr/rjaa613

**Published:** 2021-01-31

**Authors:** Maysaa Badour, Gaber Mahmoud, Ahmad Hasan, Walaa Sulaiman, Ali Hammed

**Affiliations:** Pediatric University Hospital, Division of Gastroenterology, Damascus, Syria; Pediatric University Hospital, Division of Gastroenterology, Damascus, Syria; AL-Basel Hospital, Department of Pediatric Surgery, Tartus, Syria; Pediatric University Hospital, Division of Gastroenterology, Damascus, Syria; Tishreen University Hospital, Department of Neurosurgery, Lattakia, Syria

## Abstract

Superior mesenteric artery (SMA) syndrome is a gastrovascular disorder in which the third and final portion of the duodenum is compressed between the abdominal aorta (AA) and the overlying SMA. Our case presents an 11-year-old female with chronic intermittent vomiting that started since she was 6 months old. Contrast enhanced computed tomography scan for abdomen and pelvis guided to the correct diagnosis and the patient received the adequate treatment. Awareness of a broad range of differential diagnosis of vomiting and a high degree of suspicion of SMA syndrome is fundamental in order to direct the proper diagnostic investigation. Duodenojejunostomy provides the best results in severe cases.

## INTRODUCTION

Superior mesenteric artery (SMA) syndrome is a gastrovascular disorder in which the third and final portion of the duodenum is compressed between the abdominal aorta (AA) and the overlying SMA [1].

This results in chronic, intermittent, or acute complete or partial duodenal obstruction [2]. SMA syndrome was first described in 1861 by Von Rokitansky, who proposed that its cause was obstruction of the third part of the duodenum as a result of arteriomesenteric compression. Some studies report the incidence of SMA syndrome to be 0.1–0.3% [3].

This rare, potentially life-threatening syndrome is typically caused by an angle of 6–25° between the AA and the SMA, in comparison to the normal range of 38–56°, due to a lack of retroperitoneal and visceral fat (mesenteric fat). In addition, the aortomesenteric distance is 2–8 mm, as opposed to the typical 10–20 mm [1]. However, a narrow SMA angle alone is not enough to make a diagnosis, because patients with a low body mass index, most notably children, have been known to have a narrow SMA angle with no symptoms of SMA syndrome [4].

Under normal circumstances, retroperitoneal fat and lymphatic tissues serve as a cushion, holding the SMA off the spine and protecting the duodenum from compression by it [5].

Diagnosis is challenging but should be suspected based on clinical presentation. Conservative treatment proves its effectiveness in dealing with such cases. However, failure of conservative management warrants surgical intervention.

## CASE REPORT

An 11-year-old Syrian female patient came to our clinic, complaining of severe vomiting.

She had chronic vomiting since she was a 6-month-old baby. At first, vomiting was intermittent, but in the last 3 months, it deteriorated and became more frequent, and she lost more than 6 kg over the last 2 months.

Bile-stained vomiting occurred after meals and there was no food-pain association.

She had undergone many examinations with no significant clinical findings, and failure of diagnosis.

Body examination was normal, and blood tests results (including calprotectin) were at normal ranges.

Ultrasound abdomen was done in emergency room which revealed normal findings.

Other investigations included: electroencephalogram (EEG), computed tomography (CT) of the brain and FMF mutation were also normal.

Patient was admitted to the hospital for IV hydration and gastric decompression through nasogastric tube. Four days later, an esophagogastroduodenoscopy was done, and there was large amounts of food content and dilated stomach and duodenum. The endoscopist was not able to advance the scope beyond the third part of duodenum.

Gastrographin swallow confirmed the obstruction of the third part of duodenum with proximal dilation of stomach and duodenum.

**Figure 1:**
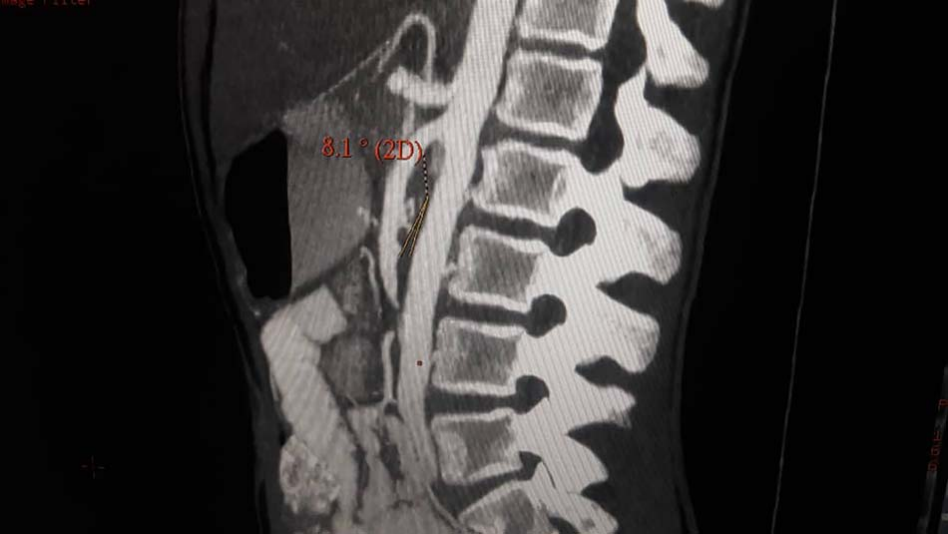
Contrast enhanced CT scan abdomen and pelvis (sagittal view) showing significant reduction of the aortomesenteric angle (measuring 8.1°) and aortomesenteric distance measuring about 7 mm.

**Figure 2:**
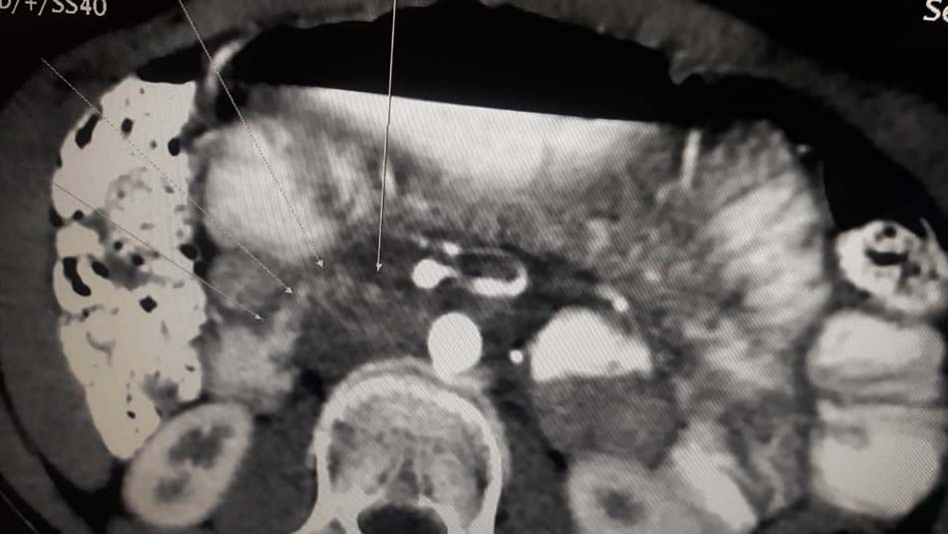
Contrast enhanced CT scan abdomen and pelvis (sagittal view) (axial view) showing duodenal compression between the aorta and superior mesenteric artery.

Patient underwent contrast enhanced CT scan for abdomen and pelvis, which revealed severely dilated stomach and significant dilatation of the duodenum up to the level of the distal third part, abrupt narrowing (transition zone) was seen just anterior to the AA and posterior to the SMA (Figs. 1 and 2) as well as significant reduction of the aortomesenteric angle (measuring 8.1°) and aortomesenteric distance measuring about 7 mm (Fig. 1).

The clinical and imaging findings of the patient concluded the diagnosis of SMA syndrome.

Due to her acute and severe symptoms (vomiting, dehydration and weight loss) the duodenojejunostomy was carried out.

Intraoperatively, we found an obvious compression on the third part of the duodenum and dilation on the first and second parts of it.

**Figure 3:**
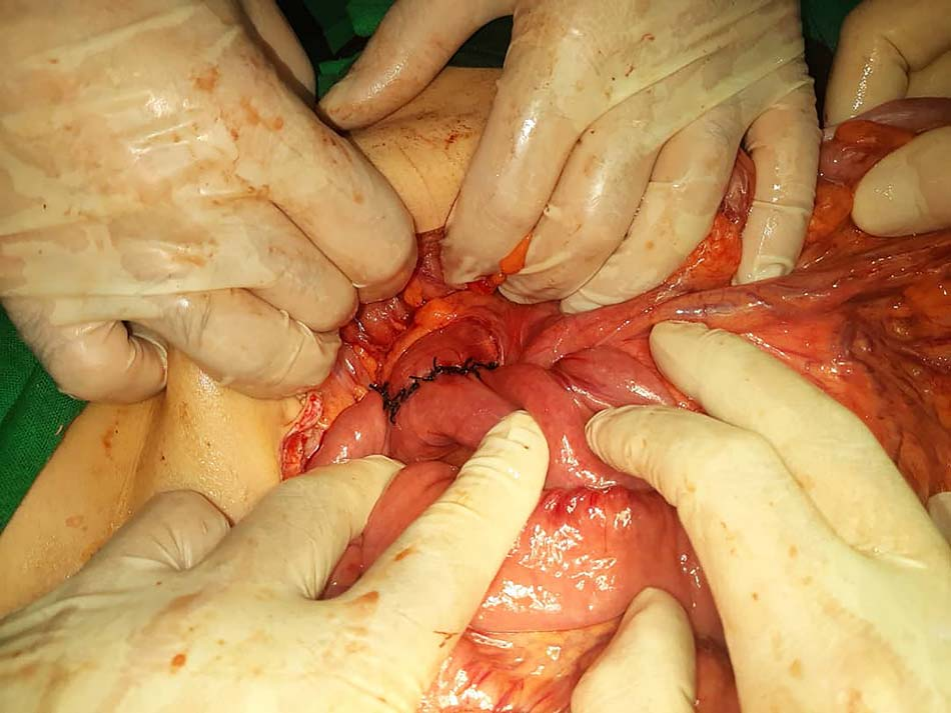
Intraoperative view of side-to-side anastomosis between duodenum and jejunum.

The procedure was a side-to-side anastomosis between the third part of the duodenum and jejunum along 15 cm from the ligament of treitz (Fig. 3).

The postoperative course was uncomplicated and the patient had a nasogastric tube for 5 days. Two weeks later, the patient started getting better and regained weight, and 3 weeks later, vomiting went away.

Twelve months medical follow-up demonstrated that she has done well with no recurrence.

## DISCUSSION

Rare causes of common symptoms often pose a diagnostic dilemma which can lead to unwanted loss of precious time in proper diagnosis and treatment.

The syndrome typically presents with signs and symptoms of upper gastrointestinal obstruction which can be mimicked by a variety of clinical conditions, such as peptic ulcer, chronic pancreatitis, mesenteric ischemia and systemic lupus erythematosus [6].

Though CT findings are highly suggestive, they can be found in normal subjects as well and, therefore, need to be interpreted in the context of individual patient’s clinical symptoms [7].

Duodenojejunostomy has been found to provide the best results in severe cases and significantly better results than gastrojejunostomy and Strong’s procedure [8].

Our case got physicians in trouble because the patient complained of intermittent vomiting for 10 years with no other red flags symptoms until the last 3 months.

Open duodenojejunostomy was performed and the patient did well postoperatively.

In conclusion, awareness of a broad range of differential diagnosis of vomiting and a high degree of suspicion for SMA syndrome are necessary in order to direct proper diagnostic investigation.

Contrast enhanced multi-detector computed tomography (MDCT) can help in an early diagnosis and rules out other causes of duodenal obstruction.

Duodenojejunostomy has been found to provide the best results in severe cases, in which conservative treatment has failed.
